# Resistance characteristics of culture-positive tuberculosis from 2015 to 2022

**DOI:** 10.3389/fpubh.2025.1543647

**Published:** 2025-05-23

**Authors:** Zhenzhen Wang, Liyang Xu, Tengfei Guo, Long Li, Qing Zhang, Jinwei Liu, Xiangyang Zu, Zhanqin Zhao, Yun Xue

**Affiliations:** ^1^The First Affiliated Hospital, College of Clinical Medicine of Henan University of Science and Technology, Luoyang, China; ^2^School of Medical Technology and Engineering, Henan University of Science and Technology, Luoyang, China; ^3^Clinical Laboratory Management Center, Luoyang Center for Disease Control and Prevention, Luoyang, Henan, China; ^4^College of Animal Science and Technology, Henan University of Science and Technology, Luoyang, China

**Keywords:** resistance, culture-positive, tuberculosis, molecular epidemiology, multicolor melting curve analysis

## Abstract

**Introduction:**

This study aimed to investigate the prevalence of resistance to first-line anti-tuberculosis (TB) drugs and the molecular mechanisms underlying resistance mutations in patients with culture-positive *Mycobacterium tuberculosis* complex (MTBC). The findings provide a data basis for developing more precise and regionally tailored anti-TB treatment regimens.

**Methods:**

From 2015 to 2022, a total of 3,605 strains isolated from 10 designated TB medical institutions in the main urban and county/township areas of Luoyang City, China, were confirmed as MTBC members through polymerase chain reaction (PCR) targeting a specific insertion sequence IS6110. Drug susceptibility testing using the proportional method was performed to analyze resistance patterns to first-line anti-TB drugs, namely, isoniazid (INH), rifampin (RFP), streptomycin (SM), and ethambutol (EMB). Molecular drug susceptibility testing was conducted on resistant strains using multicolor melting curve analysis (MMCA) to determine the mutation mechanisms associated with phenotypic resistance.

**Results:**

Among the 3,605 culture-positive MTBC cases, 79.5% (2,866 cases) were male, 64.9% (2,341 cases) resided in county and township areas, and 64.8% (2,336 cases) were younger than 60 years. The resistance rates for first-line anti-TB drugs, from highest to lowest, were SM (16.5%), INH (15.7%), RFP (9.9%), and EMB (6.4%). The overall TB resistance rates were significantly higher in the main urban areas. During the study period, the proportion of mono-resistance tuberculosis (MR-TB), multidrug-resistant tuberculosis (MDR-TB) and polydrug-resistant tuberculosis (PDR-TB) decreased by 59.2% (12.9–5.3%), 40.3% (12.4–7.4%), and 68.3% (6.9–2.2%), respectively. The predominant resistance patterns for MDR-TB and PDR-TB were MDR4 (INH + RIF + EMB + SM) and PDR2 (INH + SM). The significant molecular mutations observed were *rpsL*43 for SM resistance (66.2%, 344 cases), *katG*315 for INH resistance (70.6%, 361 cases), *rpoB*529-533 for RFP resistance (54.0%, 183 cases), and *embB*306 for EMB resistance (56.5%, 108 cases). Resistance in MDR-TB and PDR-TB cases frequently involved combinations of hotspot mutations but was not strictly confined to these sites.

**Conclusion:**

Tuberculosis resistance rates have declined over time, with distinct regional variations in resistance patterns. Significant molecular mutations responsible for drug resistance predominantly involve common hotspot mutations, but they are not limited to these.

## Introduction

Despite ongoing efforts to control tuberculosis (TB), drug-resistant TB (DR-TB) remains a significant public health challenge. DR-TB is defined as resistance to one or more anti-TB drugs (1, 2), with common subtypes including mono-resistance tuberculosis (MR-TB) (resistance to a single first-line anti-TB drug), multidrug-resistant tuberculosis (MDR-TB; resistance to at least isoniazid [INH] and rifampin [RFP]), and polydrug-resistant tuberculosis (PDR-TB; resistance to more than one first-line drug, excluding concurrent resistance to both INH and RFP), extensively drug-resistant TB (XDR-TB, resistance to INH, RFP, fluoroquinolones, and at least one second-line injectable drug), posing additional challenges to global TB control efforts (3–5). Without effective interventions, achieving the Sustainable Development Goal of ending the TB epidemic will remain difficult (6). Surveillance of DR-TB prevalence and resistance mechanisms is essential to improving TB diagnosis and treatment strategies, thereby reducing the disease's social and economic burden (7, 8). Multicolor melting curve analysis (MMCA) is widely used in China as a rapid molecular diagnostic tool for TB, providing valuable clinical treatment guidance and insights into molecular resistance mechanisms (9). However, molecular testing alone cannot fully replace *in vitro* drug susceptibility testing. Traditional TB culture and drug susceptibility testing, performed under quality assurance protocols, remain critical components of DR-TB diagnosis (10). Integrating molecular diagnostics with conventional laboratory techniques is highly beneficial for optimizing TB control strategies.

Unfortunately, local data are scarce on *in vitro* drug susceptibility patterns and corresponding molecular resistance mechanisms, which impedes efforts to implement precise TB prevention and control measures (11–13). This study investigates the resistance of culture-positive TB patients to first-line drugs in designated TB medical institutions from 2015 to 2022, analyzing molecular mutation mechanisms in conjunction with rapid molecular diagnostic techniques. The findings fill a critical gap in research on molecular drug resistance in patients with persistent bacterial excretion and provide novel insights into TB prevention and treatment. These results are expected to assist local health authorities in formulating more precise and effective policies, facilitating the transition from traditional empirical treatment to precision therapy based on molecular resistance profiles. This, in turn, is anticipated to enhance clinical treatment outcomes and mitigate the transmission risk of DR-TB.

## Methods

### Study design and data collection

**Figure 1:**
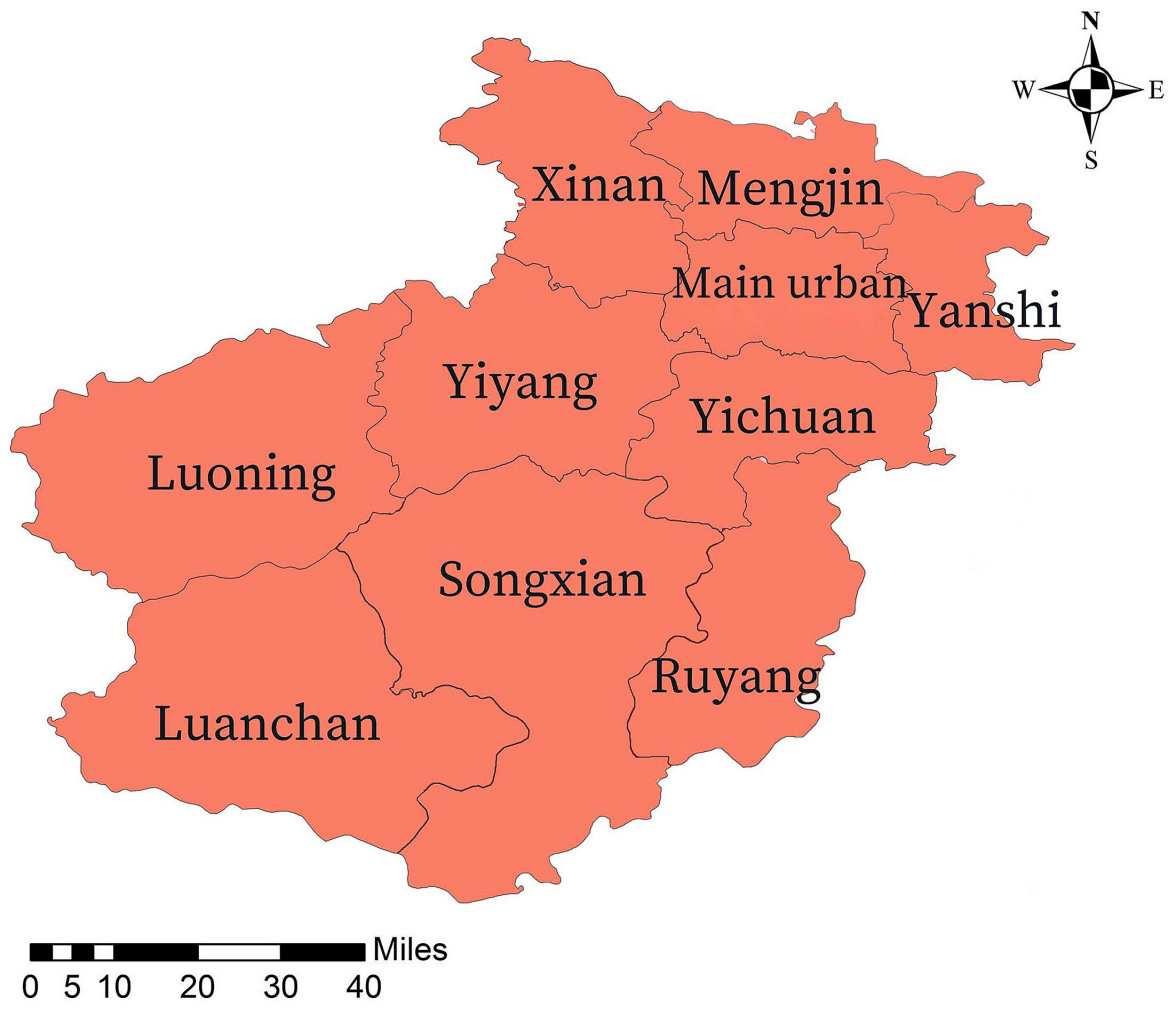
Map of the study area. One main urban area and nine county and township areas (Mengjin, Xin'an, Luanchuan, Songxian, Ruyang, Yiyang, Luoning, Yichuan, and Yanshi).

This study was conducted between January 2015 and December 2022. Sputum samples from TB patients attending 10 designated TB institutions in one main urban area and nine county and township areas of Luoyang City, China, were cultured using modified Lowenstein-Jensen medium (modified L-J medium; Henan Sainuote Biotechnology Co., Ltd., Henan, China) (Figure 1). Positive cultures underwent proportional drug susceptibility testing. MMCA was performed on phenotypically resistant MTBC isolates to assess resistance patterns to first-line anti-TB drugs and the corresponding molecular mechanisms in patients with persistent bacterial excretion. Patient demographics, including age, gender, and regional distribution, were obtained from the local TB reporting network and electronic medical record systems during diagnosis and treatment.

### Mycobacteria isolation and preliminary strain identification

Following standard guidelines for routine TB diagnosis and treatment monitoring, three consecutive sputum samples (morning, evening, and random) were collected from each patient and sent to Luoyang Infectious Disease Hospital for further analysis. The samples were smeared, acid-fast stained, examined microscopically, and recorded. Strains were isolated by culturing on Lowenstein-Jensen egg-based medium, as the International Union Against Tuberculosis and Lung Disease recommended. Small cauliflower-like colonies on the slant surface of the medium were subsequently confirmed as *Mycobacterium tuberculosis* complex (MTBC) members using p-nitrobenzoic acid (PNB) and thiophene-2-carboxylic acid hydrazide (TCH) medium tests (Henan Sainuote Biotechnology Co., Ltd., Henan, China), as well as fluorescence PCR targeting the IS6110 insertion sequence (MTBC Nucleic Acid Detection Kit, Xiamen Zeesan Biotech Co., Ltd., Xiamen, China).

The strain identification was performed using a 30-μL PCR reaction system (Xiamen Zeesan Biotech Co., Ltd., Xiamen, China) with the following thermal cycling conditions: initial uracil-DNA glycosylase (UNG) treatment at 50°C for 2 min (1 cycle) to prevent carryover contamination; pre-denaturation at 95°C for 10 min (1 cycle); touchdown PCR consisting of 10 cycles of denaturation at 95°C for 10 s, annealing starting at 71°C (decreasing by 1°C per cycle) for 15 s, and extension at 78°C for 15 s; followed by 45 amplification cycles of denaturation at 95°C for 10 s, annealing at 61°C for 15 s [with fluorescein amidite monitoring (FAM) and hexachlorofluorescein (HEX) fluorescence signal acquisition], and extension at 78°C for 15 s.

### Phenotypic susceptibility testing

Proportional drug susceptibility testing was conducted using MTBC susceptibility medium (modified L-J culture method, Henan Sainuote Biotechnology Co., Ltd., Henan, China) (14) and incubated at 36°C with 5–10% CO_2_ for 4 weeks. A colony count ratio exceeding 1% in drug-containing medium relative to drug-free control medium was considered indicative of drug resistance. The critical drug concentrations were as follows: 0.2-μg/mL isoniazid (INH), 40-μg/mL rifampicin (RFP), 2-μg/mL-ethambutol (EMB), and 4.0-μg/mL streptomycin (SM).

### Resistance gene analysis

MMCA technology enables the simultaneous detection of multiple mutation sites associated with resistance to various anti-TB drugs within a single PCR reaction system. This technique exhibits high specificity for known single-nucleotide polymorphism (SNP) loci and offers advantages such as high reproducibility, ease of operation, low cost, and minimal experimental and personnel requirements, making it particularly suitable for large-scale clinical testing. MMCA has been widely applied and validated in clinical practice (9, 15). In this study, MMCA was utilized to analyze molecular mutations associated with resistance to first-line anti-TB drugs (INH, RFP, streptomycin [SM], and EMB) in strains exhibiting resistance to at least one of these drugs in drug susceptibility testing (DST).

The genomic DNA was extracted using the boiling lysis method. First, a single loopful of MTBC colonies was scraped with a 22-standard wire gauge (22SWG) inoculation loop and suspended in 250 μL of TB DNA extraction buffer. Then, the suspension was heated at 99°C for 20 min. After that, it was centrifuged at 14,000 rpm for 10 min. Finally, the supernatant was transferred to a new 1.5-mL centrifuge tube, serving as the PCR amplification template.

The PCR protocol for molecular drug sensitivity is as follows: PCR amplification was performed in a 25-μL reaction volume (Xiamen Zeesan Biotech Co., Ltd., Xiamen, China) with the following protocol: initial UNG treatment at 50°C for 2 min (1 cycle); pre-denaturation at 95°C for 10 min (1 cycle); touchdown PCR for 10 cycles (denaturation at 95°C for 10 s, annealing starting at 71°C with 1°C decrease per cycle for 15 s; and extension at 78°C for 15 s), followed by 45 amplification cycles (denaturation at 95°C for 10 s, annealing at 61°C for 26 s with fluorescence signal acquisition and extension at 78°C for 15 s). Melting curve analysis was performed by incubation at 95°C for 2 min and 40°C for 2 min, followed by gradual heating from 40°C to 85°C at a rate of 0.04°C/s, with continuous fluorescence monitoring [FAM and Tetrachlorofluorescein (TET) channels] at 1°C intervals.

The molecular drug resistance detection kit (MMCA method), including amplification reagents, extraction reagents, and negative/positive control reagents, was obtained from Xiamen Zeesan Biotech Co., Ltd., Xiamen, China.

INH resistance (INH-R) was detected through analysis of the *ahpC* promoter region (−44 to −30 and −15 to 3 sites), *inhA94*, the *inhA* promoter region (−17 to −8 sites), and *katG315* mutations. Specific resistance sites included mutations in the INH detection region: *ahpC* promoter −44A, −42C, −39T, −34C, −32A, −30T, −15T, −12T, −10A, −10G, −10T, −9A, −6A, −4G, and *ahpC4T*; *inhA94GCG*; *inhA* promoter −17T, −16G, −15T, −11T, −8A, −8C; *katG315AGG, katG315CGC, katG315CTC, katG315ACC, katG315AAC, katG315ATC*, and *katG315ACA*. RFP resistance (RFP-R) was assessed by detecting mutations in codons 507–533 of the *rpoB* gene, encompassing the 81-base pair rifampicin resistance-determining region (RRDR). SM resistance (SM-R) was identified through mutations in codon 43 and codon 88 of the *rpsL* gene, as well as codons 513–517 and codons 905–908 of the *rrs* gene. EMB resistance (EMB-R) was evaluated by detecting mutations at codons 306, 406, and 497, as well as codons 368, 378, and 380 of the *embB* gene.

### Quality control

Two independent professionals conducted experimental procedures, data analysis, and quality control measures. The National Reference Laboratory of China regularly evaluated the laboratory. Positive control strain H37Rv (ATCC 27294) and negative control strain *Escherichia coli* (ATCC 25922) were provided by the Chinese Center for Disease Control and Prevention. Negative and positive controls were supplied with commercial kits. Standard operating procedures for drug resistance testing and instrument usage (commercial reagents and controls provided by Xiamen Zeesan Biotech Co., Ltd., Xiamen, China) were strictly followed, with intra-batch and inter-batch quality control conducted as required.

### Inclusion and exclusion criteria

The study enrolled patients who sought medical treatment at designated TB medical institutions with sputum specimens that were positive for MTBC in solid culture. Patients with negative MTBC culture results and duplicate cases of patients identified through demographic information (e.g., gender, date of birth, TB treatment history, and regional distribution) were excluded.

### Data analysis

Descriptive statistics were performed for both categorical and numerical variables. Line graphs and stacked bar charts were utilized to visualize temporal trends in the number and proportion of different resistance patterns. The Pearson chi-squared test was applied to examine associations between tuberculosis resistance patterns in the main urban area and those in county and township areas, with Fisher's exact test used as follows: when the sample size is 40 or more but 1 ≤ expected frequency < 5; when the sample size is smaller than 40; when there is any expected frequency < 1; and when the chi-squared test probability *p*-value is close to 0.05. Statistical significance was determined at a 95% confidence level, with a *p*-value of < 0.05 considered significant. All data analyses were conducted using STATA/SE 15.1 software (Stata Statistical Software: Release 15. College Station, TX, USA).

## Results

### Clinical and demographic characteristics of participants

From January 2015 to December 2022, a total of 3,605 sputum specimens from non-duplicative TB patients in 10 designated TB medical institutions in Luoyang City, Henan Province, China were successfully cultured for MTBC. Of these, 79.5% (2,866 cases) were male and 20.5% (739 cases) were female. Regarding geographic distribution, 64.9% (2,341 cases) of cases were from county and township areas, while 35.1% (1,264 cases) were from the main urban area. The age group above 60 years constituted the highest proportion at 35.2% (1,269), followed by the 41–60 years age group at 34.1% (1,229 cases). Sputum smear positive was observed in 71.7% (2,585 cases), with 61.8% (2,228 cases) graded at least 1+. The peak culture-positive rate was recorded in 2021 at 18.8% (677 cases), as shown in Table 1.

**Table 1:** Clinical and demographic characteristics of study participants (*n* = 3,605).

**Variable**	**Number *n* (%)**
**Gender**	
Male	2,866 (79.5)
Female	739 (20.5)
**Treatment**	
New cases	3,464 (96.1)
Previously treated cases	141 (3.9)
**Age category**	
< 26	441 (12.2)
26–40	666 (18.5)
41–60	1,229 (34.1)
>60	1,269 (35.2)
**Smear grade**	
Positive	2,585 (71.7)
< 9	357 (9.9)
1+	975 (27.0)
2+	652 (18.1)
3+	364 (10.1)
4+	237 (6.6)
Negative	1,020 (28.3)
**Region**	
Main urban area	1,264 (35.1)
County and township areas	2,341 (64.9)
Luanchuan	193 (5.4)
Luoning	350 (9.7)
Mengjin	296 (8.2)
Ruyang	149 (4.2)
Songxian	246 (6.8)
Xinan	176 (4.9)
Yanshi	313 (8.7)
Yichuan	260 (7.2)
Yiyang	358 (9.9)
**Year diagnosed**	
2015	233 (6.5)
2016	236 (6.5)
2017	344 (9.5)
2018	461 (12.8)
2019	539 (15.0)
2020	563 (15.6)
2021	677 (18.8)
2022	552 (15.3)

Six administrative regions (Jianxi District, Luolong District, Laocheng District, Chanhe District, Gaoxin District, and Xigong District) are collectively referred to as the main urban areas. Luanchuan, Luoning, Mengjin, Ruyang, Songxian, Xin'an, Yanshi, Yichuan, Yiyang are collectively referred to as county and township areas.

### Resistance time patterns

**Figure 2:**
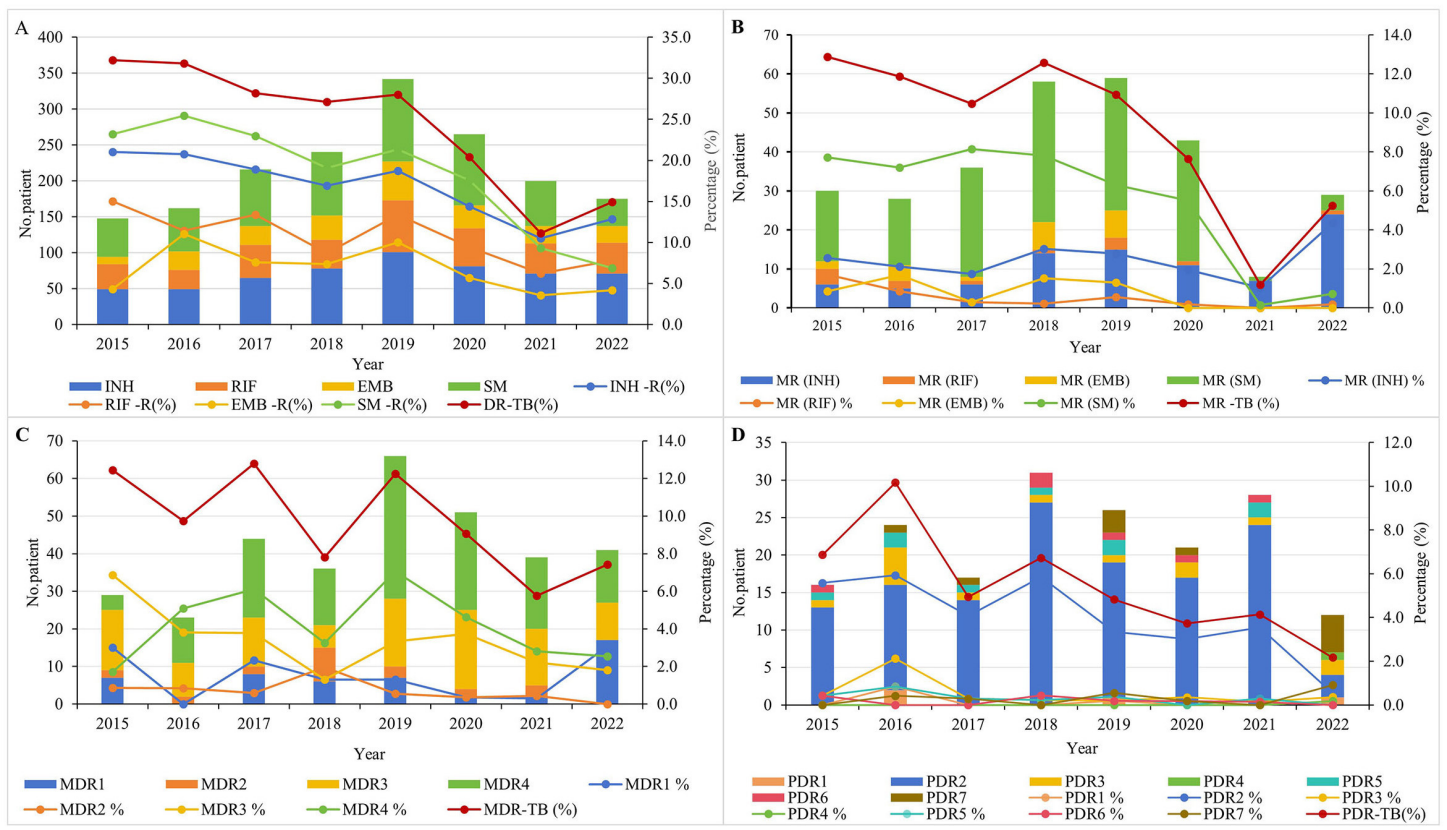
Time trend of different drug resistance patterns of tuberculosis in 2015–2022. **(A)** Time trends of first-line drug resistance for INH, RIF, EMB, and SM. **(B)** Time trends for different resistance patterns in MR-TB. **(C)** Time trends for different resistance patterns in MDR-TB. **(D)** Time trends for different resistance patterns in PDR-TB.

During the study period, the overall DR-TB detection rate in this region was 22.1% (795 cases), with a reduction of 53.7% (from 32.2 to 14.9%). The resistance rate of first-line anti-TB drugs from high to low was SM (16.5%) > INH (15.7%) > RFP (9.9%) > EMB (6.4%). The resistance rates of RFP, INH, and SM declined steadily until 2019, followed by a more rapid decline from 2019 to 2022, with overall reductions of 48.0% (from 15.0 to 7.8%), 38.6% (from 21.0 to 12.9%), and 70.3% (from 23.2 to 6.9%), respectively. The EMB resistance rate increased from 4.3% in 2015, peaked at 10.0% in 2019, and then dropped to 4.2% in 2022, showing a marginal overall decline of 2.3%. Between 2015 and 2020, SM-R and INH-R were the predominant resistance patterns; however, from 2020 to 2022, the prevalence of SM-R declined, while INH-R and RFP-R increased, ultimately making INH-R and RFP-R the most common resistance patterns by the end of 2022 (Figure 2A). The detection rate of MR-TB was 8.1% (291 cases), with 59.2% reduction (from 12.9% in 2015 to 5.3% in 2022). Of which, the decline rate of MR-TB (SM) was the most remarkable, reaching 90.9% (from 7.7% in 2015 to 0.7% in 2022). Conversely, MR-TB (INH) showed an increasing trend, rising by 65.4% (from 2.6% in 2015 to 4.3% in 2022) (Figure 2B). The detection rate of MDR-TB was 9.1% (329 cases), and declined by 40.3% (from 12.4% in 2015 to 7.4% in 2022). In 2015, MDR3 (INH + RIF + SM) was the predominant pattern (6.9%), followed by a rapid decline. During 2016–2021, MDR4 (INH + RIF + EMB + SM) was predominant. However, after 2019, its prevalence decreased, and MDR1 (INH + RIF) became the most common pattern by 2022 (Figure 2C). The PDR-TB detection rate was 4.9% (179 cases), and seven PDR-TB patterns were observed, with PDR2 (INH + SM) being the most prevalent (3.2%), followed by PDR3 (INH + EMB + SM) at 0.4% and PDR7 (EMB + SM) at 0.3%. The overall detection rate of PDR-TB declined by 68.3% (from 6.9% in 2015 to 2.2% in 2022), with PDR2 (INH + SM) decreasing more substantially by 91.1% (from 5.6% in 2015 to 0.5% in 2022). By 2022, PDR7 (EMB + SM) was the dominant pattern, accounting for 0.9% (Figure 2D).

### Spatial pattern of drug resistance

Significant differences in detection rates were observed between the main urban area and county/township areas for INH-R, RFP-R, EMB-R, SM-R, MDR-TB, and PDR-TB (*p* < 0.001 for all comparisons except PDR-TB, which was *p* = 0.017). No significant difference was found in the detection rates of MR-TB (*p* = 0.801). The main urban area had a drug-resistant TB detection rate of 26.8% (339 cases), whereas Ruyang, Luoning, Xin'an, and Yiyang exhibited relatively high rates of 23.5% (35 cases), 22.9% (80 cases), 22.2% (39 cases), and 21.5% (77 cases), respectively. Ruyang had the highest MR-TB rate (10.7%, 16 cases), followed by Luoning (10.0%, 35 cases), Yiyang (9.5%, 34 cases), the main urban area (8.2%, 104 cases), and Songxian with the lowest rate (6.1%, 15 cases). Xin'an (9.7%, 17 cases), Luoning (9.1%, 32 cases), Ruyang (8.7%, 13 cases), and Songxian (8.1%, 20 cases) had relatively high MDR-TB detection rates. Regarding PDR-TB, the highest detection rates were observed in Songxian (5.7%, 14 cases), Yiyang (5.3%, 19 cases), Luanchuan (5.2%, 10 cases), and Mengjin (5.1%, 15 cases), while Yanshi had the lowest (1.9%, 6 cases). The distribution of MDR patterns varied, with MDR4 (INH + RFP + EMB + SM) being predominant in the main urban area, Luoning, Songxian, Luanchuan, Yiyang, and Yichuan. However, in Mengjin, Ruyang, and Xin'an, MDR3 (INH + RFP + SM) was the predominant pattern. PDR patterns also varied regionally, with PDR2 (INH + SM) identified exclusively in Luanchuan and Ruyang, and a single case of PDR4 (RFP + EMB) detected in Xin'an (Table 2).

**Table 2:** Different resistance patterns of MTBC distributed by region.

**Resistance pattern**	**Total^a^**	**County and township areas**										**Main urban**	***p*-vaule**
		**Luanchuan**	**Luoning**	**Mengjin**	**Ruyang**	**Songxian**	**Xin'an**	**Yanshi**	**Yichuan**	**Yiyang**	**Total** ^b^		
	***n*** **(%)**	***n*** **(%)**	***n*** **(%)**	***n*** **(%)**	***n*** **(%)**	***n*** **(%)**	***n*** **(%)**	***n*** **(%)**	***n*** **(%)**	***n*** **(%)**	***n*** **(%)**	***n*** **(%)**	
DR-TB	795 (22.1)	37 (19.2)	80 (22.9)	55 (18.6)	35 (23.5)	49 (19.9)	39 (22.2)	44 (14.1)	40 (15.4)	77 (21.5)	456 (19.5)	339 (26.8)	< 0.001^*^
**Any resistance to first-line drugs**													
INH	565 (15.7)	27 (14.0)	58 (16.6)	37 (12.5)	22 (14.8)	34 (13.8)	27 (15.3)	27 (8.6)	26 (10.0)	45 (12.6)	303 (12.9)	262 (20.7)	< 0.001^*^
RIF	358 (9.9)	15 (7.8)	36 (10.3)	19 (6.4)	15 (10.1)	20 (8.1)	19 (10.8)	19 (6.1)	15 (5.8)	29 (8.1)	187 (8.0)	171 (13.5)	< 0.001^*^
EMB	229 (6.4)	9 (4.7)	23 (6.6)	8 (2.7)	9 (6.0)	19 (7.7)	6 (3.4)	13 (4.2)	8 (3.1)	23 (6.4)	118 (5.0)	111 (8.8)	< 0.001^*^
SM	596 (16.5)	27 (14.0)	53 (15.1)	45 (15.2)	24 (16.1)	38 (15.4)	27 (15.3)	28 (8.9)	32 (12.3)	59 (16.5)	333 (14.2)	263 (20.8)	< 0.001^*^
MR-TB	291 (8.1)	13 (6.7)	35 (10.0)	21 (7.1)	16 (10.7)	15 (6.1)	14 (8.0)	21 (6.7)	18 (6.9)	34 (9.5)	187 (8.0)	104 (8.2)	0.801
INH	88 (2.4)	3 (1.6)	16 (4.6)	4 (1.4)	3 (2.0)	3 (1.2)	3 (1.7)	6 (1.9)	5 (1.9)	8 (2.2)	51 (2.2)	37 (2.9)	0.165
RIF	13 (0.4)	1 (0.5)	2 (0.6)	0 (0.0)	2 (1.3)	0 (0.0)	1 (0.6)	1 (0.3)	0 (0.0)	1 (0.3)	8 (0.3)	5 (0.4)	0.778
EMB	21 (0.6)	2 (1.0)	0 (0.0)	1 (0.3)	3 (2.0)	2 (0.8)	0 (0.0)	4 (1.3)	0 (0.0)	3 (0.8)	15 (0.6)	6 (0.5)	0.532
SM	169 (4.7)	7 (3.6)	17 (4.9)	16 (5.4)	8 (5.4)	10 (4.1)	10 (5.7)	10 (3.2)	13 (5.0)	22 (6.1)	113 (4.8)	56 (4.4)	0.591
MDR-TB	329 (9.1)	14 (7.3)	32 (9.1)	19 (6.4)	13 (8.7)	20 (8.1)	17 (9.7)	17 (5.4)	14 (5.4)	24 (6.7)	170 (7.3)	159 (12.6)	< 0.001^*^
MDR1 (INH + RIF)	49 (1.4)	4 (2.1)	7 (2.0)	5 (1.7)	0 (0.0)	2 (0.8)	5 (2.8)	4 (1.3)	2 (0.8)	4 (1.1)	33 (1.4)	16 (1.3)	0.722
MDR2 (INH + RIF + EMB)	23 (0.6)	0 (0.0)	1 (0.3)	0 (0.0)	3 (2.0)	4 (1.6)	2 (1.1)	1 (0.3)	0 (0.0)	1 (0.3)	12 (0.5)	11 (0.9)	0.198
MDR3 (INH + RIF + SM)	108 (3.0)	3 (1.6)	6 (1.7)	9 (3.0)	7 (4.7)	5 (2.0)	7 (4.0)	6 (1.9)	5 (1.9)	8 (2.2)	56 (2.4)	52 (4.1)	0.004^*^
MDR4 (INH + RIF + EMB + SM)	149 (4.1)	7 (3.6)	18 (5.1)	5 (1.7)	3 (2.0)	9 (3.7)	3 (1.7)	6 (1.9)	7 (2.7)	11 (3.1)	69 (2.9)	80 (6.3)	< 0.001^*^
PDR-TB	175 (4.9)	10 (5.2)	13 (3.7)	15 (5.1)	6 (4.0)	14 (5.7)	8 (4.5)	6 (1.9)	8 (3.1)	19 (5.3)	99 (4.2)	76 (6.0)	0.017^*^
PDR1 (INH + EMB)	4 (0.1)	0 (0.0)	1 (0.3)	0 (0.0)	0 (0.0)	0 (0.0)	0 (0.0)	0 (0.0)	1 (0.4)	1 (0.3)	3 (0.1)	1 (0.1)	/
PDR2 (INH + SM)	130 (3.6)	10 (5.2)	7 (2.0)	13 (4.4)	6 (4.0)	10 (4.1)	7 (4.0)	4 (1.3)	6 (2.3)	8 (2.2)	71 (3.0)	59 (4.7)	0.012^*^
PDR3 (INH + EMB + SM)	14 (0.4)	0 (0.0)	2 (0.6)	1 (0.3)	0 (0.0)	1 (0.4)	0 (0.0)	0 (0.0)	0 (0.0)	4 (1.1)	8 (0.3)	6 (0.5)	0.580
PDR4 (RIF + EMB)	1 (0.0)	0 (0.0)	0 (0.0)	0 (0.0)	0 (0.0)	0 (0.0)	1 (0.6)	0 (0.0)	0 (0.0)	0 (0.0)	1 (0.0)	0 (0.0)	/
PDR5 (RIF + SM)	9 (0.2)	0 (0.0)	2 (0.6)	0 (0.0)	0 (0.0)	0 (0.0)	0 (0.0)	0 (0.0)	1 (0.4)	3 (0.8)	6 (0.3)	3 (0.2)	/
PDR6 (RIF + EMB + SM)	6 (0.2)	0 (0.0)	0 (0.0)	0 (0.0)	0 (0.0)	0 (0.0)	0 (0.0)	1 (0.3)	0 (0.0)	1 (0.3)	2 (0.1)	4 (0.3)	0.193
PDR7 (EMB + SM)	11 (0.3)	0 (0.0)	1 (0.3)	1 (0.3)	0 (0.0)	3 (1.2)	0 (0.0)	1 (0.3)	0 (0.0)	2 (0.6)	8 (0.3)	3 (0.2)	0.757

^a^Total represent the total number of TB cases in the county and township areas as well as the main urban area.

^b^Total represent the total number of TB cases in all county and township areas; *p*-value, shows the differences in detection of drug resistance between main urban areas and overall county and township areas.

^*^Significant statistical difference. In this study, the significance level is set at *p* < 0.05.

### Molecular mutation mechanisms identified by multicolor melting curve analysis

The molecular resistance rates for INH, RFP, SM, and EMB were 90.4% (511/565), 94.7% (339/358), 87.2% (520/596), and 83.4% (191/229), respectively. INH-R was primarily attributed to *KatG315* alterations, accounting for 70.6% (361 cases), including mutations at 62.8% (321 cases) and deletions at 7.8% (40 cases). Additional mutations were identified in the *inhA* promoter region (−17 to −8 sites) at 15.7% (80 cases), and the *ahpC* promoter region (−44 to −30 and −15 to 3 sites) at 4.9% (25 cases). RFP-R was predominantly due to *rpoB*529-533 mutations (54.0%, 183 cases), followed by mutations in *rpoB*521-528 (18.9%, 64 cases), *rpoB*513-520 (10.3%, 35 cases), and *rpoB*507-512 (7.1%, 24 cases). SM-R was primarily associated with *rpsL*43 mutations (66.2%, 344 cases), with additional mutations observed in *rpsL*88 (18.7%, 97 cases) and *rrs*513-517 (6.3%, 33 cases). EMB-R was linked to mutations in *embB*306 (56.5%, 108 cases), *embB*406 (24.1%, 46 cases), and *embB*497 (15.2%, 29 cases). MR-TB (SM) was predominantly caused by mutations in *rpsL*43 (37.9%, 99 cases), *rpsL*88 (9.2%, 24 cases), and *rrs*513-517 (6.1%, 16 cases). MR-TB (INH) was mainly attributed to mutations in *KatG*315 (18.0%, 47 cases) and the *inhA* promoter region (−17 to −8 sites) (4.6%, 12 cases). MR-TB (EMB) and MR-TB (RFP) were due to mutation in *embB*306 (3.8%, 10 cases) and *rpoB*529-533 (2.7%, 7 cases), respectively. The primary molecular mechanisms underlying MDR-TB involved combined mutations in *KatG*315 & *rpoB*529-533 & *embB*306 & *rpsL*43 (13.6%, 40 cases), *KatG*315 & *rpoB*529-533 & *rpsL*43 (10.5%, 31 cases), and *KatG*315 & *rpoB*529-533 & *embB*406 & *rpsL*88 (9.8%, 29 cases). The predominant mechanism for PDR-TB was the combined mutation of *KatG*315 & *rpsL*43 (60.3%, 94 cases) (Table 3).

**Table 3:** Molecular mechanisms of different resistance types.

**Resistance pattern**	**Detectable region or mutation sites**	**Frequency, *n* (%)**
Any resistance to first-line drugs		1,561 (43.3)
INH		511 (32.7)
	*KatG*315	321 (62.8)
	*inhA* promoter region (−17 ~−8 site)	80 (15.7)
	*KatG*315 codon delate	40 (7.8)
	*ahpC* promoter region (−44 ~−30 and −15 ~ 3 sites)	25 (4.9)
	*inhA*94	16 (3.1)
	*inhA* promoter region (−17 ~−8 site) & *KatG*315	12 (2.3)
	*ahpC* promoter region (−44 ~−30 and −15 ~ 3 sites) & *KatG*315	9 (1.8)
	*ahpC* promoter region (−44 ~−30 and −15 ~ 3 sites) & *inhA94*	3 (0.6)
	*ahpC* promoter region (−44 ~−30 and −15 ~ 3 sites) & *inhA* promoter region (−17 ~−8 site)	2 (0.4)
	*inhA*94 & *KatG*315	2 (0.4)
	*inhA94* & *inhA* promoter region (−17 ~−8 site)	1 (0.2)
RFP		339 (21.7)
	*rpoB*529-533	183 (54.0)
	*rpoB*521-528	64 (18.9)
	*rpoB*513-520	35 (10.3)
	*rpoB*507-512	24 (7.1)
	*rpoB*521-528 & *rpoB*529-533	10 (2.9)
	*rpoB*513-520 & *rpoB*529-533	7 (2.7)
	*rpoB*513-520 & *rpoB*521-528	7 (2.1)
	*rpoB*507-512 & *rpoB*513-520	5 (1.5)
	*rpoB*507-512 & *rpoB*513-520 & *rpoB*521-528	2 (0.6)
	*rpoB*507-512 & *rpoB*529-533	1 (0.3)
	*rpoB*507-512 & *rpoB*521-528	1 (0.3)
SM		520 (33.3)
	*rpsL*43	344 (66.2)
	*rpsL*88	97 (18.7)
	*rrs*513-517	33 (6.3)
	*rrs*905-908	17 (3.3)
	*rpsL*88 & *rrs*905-908	11 (2.1)
	*rpsL*43 & *rrs*513-517	9 (1.7)
	*rpsL*43 & *rrs*905-908	6 (1.2)
	*rrs*513-517 & *rrs*905-908	3 (0.6)
EMB		191 (12.2)
	*embB*306	108 (56.5)
	*embB*406	46 (24.1)
	*embB*497	29 (15.2)
	*embB*368/378/380	8 (4.2)
MR-TB		261 (7.2)
INH		76 (29.1)
	*KatG*315	47 (18.0)
	*inhA* promoter region (−17 ~−8 site)	12 (4.6)
	*KatG*315 codon delate	7 (2.7)
	*ahpC* promoter region (−44 ~−30 and −15 ~ 3 sites)	4 (1.5)
	*inhA94*	3 (1.1)
	*ahpC* promoter region (−44 ~−30 and −15 ~ 3 sites) & *KatG*315	1 (0.4)
	*inhA* promoter region (−17 ~−8 site) & *KatG*315	1 (0.4)
	*ahpC* promoter region (−44 ~−30 and −15 ~ 3 sites) & *inhA* promoter region (−17 ~−8 site)	1 (0.4)
RFP		12 (4.6)
	*rpoB*529-533	7 (2.7)
	*rpoB*521-528	2 (0.8)
	*rpoB*507-512	1 (0.4)
	*rpoB*513-520	1 (0.4)
	*rpoB*521-528 & *rpoB*529-533	1 (0.4)
SM		155 (59.4)
	*rpsL*43	99 (37.9)
	*rpsL*88	24 (9.2)
	*rrs*513-517	16 (6.1)
	*rrs*905-908	6 (2.3)
	*rpsL*43 & *rrs*513-517	5 (1.9)
	*rpsL*43 & *rrs*905-908	2 (0.8)
	*rpsL*88 & *rrs*905-908	2 (0.8)
	*rrs*513-517 & *rrs*905-908	1 (0.4)
EMB		18 (6.9)
	*embB*306	10 (3.8)
	*embB*406	5 (1.9)
	*embB*497	2 (0.8)
	*embB*368/378/380	1 (0.4)
MDR		295 (8.2)
MDR1 (INH + RIF)		44 (14.9)
	*KatG*315 & *rpoB*529-533	20 (6.8)
	*inhA* promoter region (−17 ~−8 site) & *rpoB*529-533	12 (4.1)
	*inhA* promoter region (−17 ~−8 site) & *rpoB*521-528 & *rpoB*529-533	4 (1.4)
	*ahpC* promoter region (−44 ~−30 and −15 ~ 3 sites) & *rpoB*529-533	4 (1.4)
	*KatG*315 & *rpoB*513-520	2 (0.7)
	*KatG*315 & *rpoB*521-528 & *rpoB*529-533	1 (0.3)
	*KatG*315 codon delate & *rpoB*507-512	1 (0.3)
MDR2 (INH + RIF + EMB)		21 (7.1)
	*KatG*315 & *rpoB*529-533 & *embB*306	12 (4.1)
	*inhA* promoter region (−17 ~−8 site) & *rpoB*529-533 & *embB*306	6 (2.0)
	*KatG*315 & *rpoB*507-512 & *embB*497	2 (0.7)
	*inhA* promoter region (−17 ~−8 site) & *rpoB*529-533 & *embB*368/378/380	1 (0.3)
MDR3 (INH + RIF + SM)		97 (32.9)
	*KatG*315 & *rpoB*529-533 & *rpsL*43	35 (11.9)
	*inhA* promoter region (−17 ~−8 site) & *rpoB*529-533 & *rpsL*43	22 (7.5)
	*KatG*315 & *rpoB*529-533 & *rpsL*88	12 (4.1)
	*KatG*315 & *rpoB*521-528 & *rpsL*43	10 (3.4)
	*KatG*315 & *rpoB*507-512 & *rpsL*43	7 (2.4)
	*KatG*315 & *rpoB*521-528 & *rpsL*88	6 (2.0)
	*KatG*315 & *rpoB*507-512 & *rpoB*521-528 & *rpsL*43	3 (1.0)
	*KatG*315 & *rpoB*529-533 & *rrs*905-908	1 (0.3)
	*ahpC* promoter region (−44 ~−30 and −15 ~ 3 sites) & *rpoB*521-528 & *rrs*513-517	1 (0.3)
MDR4 (INH + RIF + EMB + SM)		133 (45.1)
	*KatG*315 & *rpoB*529-533 & *embB*306 & *rpsL*43	40 (13.6)
	*KatG*315 & *rpoB*529-533 & *embB*406 & *rpsL*88	29 (9.8)
	*KatG*315 & *rpoB*529-533 & *embB*306 & *rrs*513-517	20 (6.8)
	*KatG*315 & *rpoB*513-520 & *embB*306 & *rpsL*43	18 (6.1)
	*KatG*315 & *rpoB*521-528 & *embB*406 & *rpsL*43	12 (4.1)
	*KatG*315 & *rpoB*513-520 & *embB*406 & *rpsL*43	10 (3.4)
	*ahpC* promoter region (−44 ~−30 and −15 ~ 3 sites) & *KatG*315 & *rpoB*529-533 & *embB*306 & *rpsL*43	2 (0.7)
	*KatG*315 & *rpoB*507-512 & *embB*406 & *rpsL*43	1 (0.3)
	*KatG*315 & *rpoB*507-512 & *rpoB*529-533 & *embB*406 & *rpsL*88	1 (0.3)
PDR		156 (4.3)
PDR1 (INH + EMB)		4 (2.6)
	*KatG*315 & *embB*306	3 (1.9)
	*inhA* promoter region (−17 ~−8 site) & *embB*406	1 (0.6)
PDR2 (INH + SM)		116 (74.4)
	*KatG*315 & *rpsL*43	94 (60.3)
	*inhA* promoter region (−17 ~−8 site) & *rpsL*43	8 (5.1)
	*KatG*315 & *rrs*513-517	4 (2.6)
	*ahpC* promoter region (−44 ~−30 and −15 ~ 3 sites) & *rpsL*43	4 (2.6)
	*inhA* promoter region (−17 ~−8 site) & *KatG*315 & *rpsL*43	2 (1.3)
	*KatG*315 & *rpsL*43 & *rrs*513-517	2 (1.3)
	*inhA* promoter region (−17 ~−8 site) & *rrs*905-908	1 (0.6)
	*KatG*315 & *rpsL*43 & *rrs*905-908	1 (0.6)
PDR3 (INH + EMB + SM)		12 (7.7)
	*KatG*315 & *embB*406 & *rpsL*43	6 (3.8)
	*KatG*315 & *embB*306 & *rrs*513-517	3 (1.9)
	*KatG*315 & *embB*497 & *rpsL*43	2 (1.3)
	*inhA94* & *KatG*315 & *embB*406 & *rpsL*88	1 (0.6)
PDR4 (RIF + EMB)		1 (0.6)
	*rpoB*513-520 & *embB*406	1 (0.6)
PDR5 (RIF + SM)		8 (5.1)
	*rpoB*529-533 & *rpsL*43	4 (2.6)
	*rpoB*513-520 & *rpsL*43	2 (1.3)
	*rpoB*513-520 & *rpsL*43 & *rrs*513-517	1 (0.6)
	*rpoB*513-520 & *rpoB*529-533 & *rpsL*43	1 (0.6)
PDR6 (RIF + EMB + SM)		5 (3.2)
	*rpoB*529-533 & *emb*306 & *rpsL*43	2 (1.3)
	*rpoB*521-528 & *rpoB*529-533 & *embB*406 & *rpsL*88	1 (0.6)
	*rpoB*529-533 & *embB*497 & *rpsL*43	1 (0.6)
	*rpoB*513-520 & *embB*406 & *rpsL*88	1 (0.6)
PDR7 (EMB + SM)		10 (6.4)
	*embB*406 & *rpsL*88	8 (5.1)
	*embB*306 & *rrs*513-517	1 (0.6)
	*embB*368/378/380 & *rrs*905-908	1 (0.6)

## Discussion

TB is a globally prevalent respiratory disease caused by MTBC and poses a significant threat to human health. In 2022, ~10.6 million new TB cases were reported worldwide, with 7.4% occurring in China (4). In the same year, TB resulted in 1.3 million deaths among HIV-negative individuals globally, including ~33,000 fatalities in China (4). To date, TB remains the leading cause of mortality from a single infectious agent. The influence of gender on TB susceptibility remains a subject of debate. Data demonstrate the male-to-female ratio (M/F ratio) of TB incidence ranges from 1.2:1 to 4.9:1, with consistently higher ratios observed in Asian populations compared to African cohorts (16). In Ethiopia, Nigeria, Sudan, Uganda, and other regions, TB prevalence is highest among individuals aged 35–54 years. In contrast, in some parts of China, the incidence among individuals aged 65 years is twice that observed in younger populations (17). Rural areas bear the most significant TB burden, mainly due to disparities in economic development (18, 19). In the present study, culture-positive TB incidence was 3.9 times higher in males than females, 1.9 times higher in county and township areas than in the main urban area, and 2.3 times higher in individuals over 40 years than in those under 40 years. These findings align with our previous reports on rapid molecular TB detection (12). The observed gender-related differences may be attributed to both biological factors (e.g., differences in lung physiology, immune system regulation by sex hormones, and genetic susceptibility) and sociobehavioral factors (e.g., lifestyle, occupation, and socioeconomic status) (20).

The overall detection rate of DR-TB exhibited a declining trend from 2015 to 2022. Prior to 2019, variations in TB resistance patterns remained relatively stable; however, a sharp decline occurred thereafter. This situation aligns closely with the overall reduction in TB incidence in China over the past decade (13). Overall, the observed decline in drug resistance rates results from sustained efforts in TB control, validating the effectiveness of anti-TB strategies and highlighting the impact of prevention and control policies. Indeed, the widespread adoption of rapid molecular diagnostic techniques, extensive public health education and awareness campaigns, expanded medical insurance coverage, and the global TB control agenda have all contributed to progress in local TB control efforts. However, the COVID-19 pandemic in 2019 led to a significant diversion of medical resources, hindering timely TB treatment and case reporting (21). Consequently, the observed decline in TB resistance may not accurately reflect the actual situation, necessitating our close attention and an objective approach (22). Some studies suggest that, to achieve a phased victory in TB control, the annual average decline in TB incidence in China from 2022 to 2025 must reach 14.5%, thereby contributing to the realization of the “End TB Strategy” by 2035 (4, 13). However, the average annual decline rate of TB resistance in this region is only 9.8%, presenting substantial challenges to local TB control efforts. Moreover, an in-depth analysis of local resistance patterns indicates their prevalence has evolved. So, the decline of phenotypic resistance rate cannot be used as the only indicator to measure the effectiveness of local anti-TB (23, 24). DR-TB management remains a formidable challenge, necessitating the formulation of more precise and effective policies, the implementation of targeted prevention and control measures tailored to specific resistance patterns, and the intensification of efforts to contain DR-TB transmission in the post-COVID-19 era.

MTBC continues to circulate locally, exhibiting significant regional variations in distribution and resistance patterns. Phenotypic drug resistance testing for SM and INH revealed relatively high resistance rates of 16.5 and 15.7%, respectively. This may be attributed to the prolonged use of these two drugs in first-line anti-TB regimens, underscoring the need to further strengthen Directly Observed Therapy Shortcourse (DOTS) regimens to better control TB resistance (11, 13). The local TB resistance rate (22.1%) is lower than the national drug resistance baseline survey estimate (39.12%) but higher than those reported in Shandong Province (18.7%) and Jilin City (12.24%), and comparable to that of Shanghai (21%) (8, 16, 25, 26). The local multi-drug resistance rate (9.1%) exceeds that of the aforementioned three regions (3.22%, 8.16%, and 4.98%) as well as the national average (4.5%) (26, 27). These findings suggest there remains substantial room for improvement in DR-TB management. The geographical distribution of the TB burden in China is heterogeneous, with a higher disease burden observed in the western regions (13). Variability in TB distribution across provinces is also evident; for example, TB cases in Xinjiang are predominantly concentrated in the southern region, whereas in Tibet, cases are primarily found in the southeastern areas (28). Locally, resistance rates were highest in main urban areas (see Table 2). In China, due to the nature of the medical insurance system, household registration status has a limited impact on the treatment outcomes of confirmed TB patients. TB patients from different residential areas often converge at specific hospitals for standardized treatment (29), leading to the concentration of complex and difficult-to-treat cases in urban areas, which may account for the higher TB resistance rates observed in these areas. Additionally, factors such as high mental stress, low BMI, caused by societal influences (e.g., aesthetic preferences in urban populations), are also associated with an increased risk of TB resistance (30). In recent years, the rapid development of tourism in Luoyang has significantly contributed to the local economy, attracting a substantial influx of migrants. This increased mobility facilitates the rapid spread of TB, and poor treatment adherence among migrants is a critical factor contributing to high TB resistance (31). The regional disparities in phenotypic drug resistance are potentially influenced by variations in treatment regimens, the intensity of control measures, the intrinsic resistance of MTBC, and exposure to drug-resistant infection sources. Luoyang, situated in western Henan Province, is an industrial city characterized by hilly terrain and an underdeveloped economy. The diverse industrial structures and demographic profiles in different areas also contribute to regional variations in drug resistance. Therefore, developing targeted control strategies, rationally allocating resources, strengthening regional cooperation and information sharing, and collaboratively addressing the challenge of TB drug resistance remain key priorities for TB control efforts.

Rapid detection based on molecular biology provides essential support for the early diagnosis and treatment of drug-resistant TB. Molecular resistance characteristics result from the interplay of multiple factors. This study identified 1,561 strains with molecular mutation among 1,748 phenotypically resistant MTBC strains (Tables 2, 3), indicating that the local TB resistance mechanism is complex. In addition to common molecular mutations, other resistance mechanisms or non-hotspot mutations contribute to the spread of drug-resistant tuberculosis in the region. Mutations in target genes or regulatory regions of antimicrobial agents are highly correlated with TB resistance. Monitoring these hotspot mutations remains clinically valuable for predicting TB resistance, even though molecular resistance does not fully represent phenotypic resistance (32, 33).

Mutations in *katG*315, *rpoB*529-533, *rpsL*43, and *embB*306 were highly associated with INH-R, RFP-R, SM-R, and EMB-R, respectively, in this region, which represents the most common hotspot mutations of TB resistance. However, regional differences exist in the location and frequency of these gene mutations. The detection rate of INH-R caused by *katG*315 mutations varies globally from 42 to 90% (34). Locally, this rate was 75.1% (384 cases), with 70.6% (361 cases) attributable to independent mutations at the *katG*315 site. This proportion is significantly higher than that reported in Japan and Italy (28–38%), Western Africa and Spain (43–46%), and southern India and Australia (61–66%), but lower than that in Eastern Europe, Russia, and South Africa (86–97%), while being similar to that in Switzerland (71%) (35–37). A high detection rate of *katG*315 mutations suggests that molecular diagnostic techniques targeting this site can be effectively utilized for the rapid screening of INH-R strains and early intervention in patients. In general, mutations in the *inhA* promoter region occur in 12–42% of INH-R strains (38). The mutation proportion in the *inhA* promoter region (−17 to −8 sites) locally is 15.7% (80 cases), which is consistent with the national average in China (15.2%) (39) but lower than that in India (28.1%) (37) and Canada (35.56%) (40), while higher than that in Uganda, Poland and Kyrgyz Republic (3.7–7%) (37). Additionally, the presence of mutations in other genes and combined mutations suggests that addressing the complex challenge of INH-R requires consideration of heterogeneous resistance in local DR-TB management and control. Reports indicate that more than 96.0% of RFP resistance is due to mutations in the RRDR of the *rpoB* gene (41). Among these, single-site mutations make up 87.3%, two-site mutations account for 5.1%, and multisite combined mutations are 0.3% (42). These findings closely align with our data, which identified RRDR variations in 94.7% of RFP-R strains, with single-, two-, and multisite mutations occurring at 90.3%, 8.8%, and 0.9%, respectively. However, regional variations persist. For example, the mutation rate of the RRDR in Wuhan reaches 97.93% (41), 86.1% in Africa, and 76.4% in Iran (43). Locally, SM-R due to mutations in *rpsL*43 accounts for 66.2%, which is comparable to findings from Zhejiang (65.0%) (39) and Guangzhou (67.15%) (41, 44, 45), higher than in Yunnan (54.2%) (46) but lower than in Beijing (72.0%) (47) and Tianjin (81.13%) (48). Compared with foreign studies, it is higher than that in North India (48.9%) (49) and South Korea (52.8%) (50), and lower than that in Poland (71.9%) and Singapore (80.4%) (51). The *embB* gene, particularly the *embB*306 locus, is highly correlated with EMB-R (52). The local *embB*306 mutation rate (56.5%) exceeds that reported in Mexico (33.0%) and Russia (48.28%), but is lower than in Germany (68.0%) and Hunan (71.4%), and comparable to findings in Hebei (56.45%) and France (56.34%) (53–55). The emergence of local MDR-TB and PDR-TB primarily results from combinations of mutations in single anti-TB drug resistance hotspot genes, though additional mechanisms may be involved. Notably, MDR-TB presents a particularly complex drug resistance mechanism, highlighting its critical role in TB management. The resistance mechanisms of MTBC are intricate, with interactions among resistance sites following a linkage mechanism. Resistance outcomes are influenced by the gene environment in which mutations occur and by concurrent mutations in other resistance-associated loci. For example, the *embB*306 mutation significantly contributes to the development of RIF-R and INH-R, thereby promoting MDR-TB. Moreover, resistance-related mutations are also observed in susceptible strains, reflecting polymorphisms in evolutionary processes (56, 57).

These regional variations in resistance mutation sites underscore the necessity of formulating precise prevention and control strategies and personalized treatment plans. Furthermore, they provide direction for developing advanced drug resistance detection technologies, underscoring the global challenges in TB control. Sharing resistance gene data across countries is essential to enhance the global understanding of TB resistance trends and facilitate collaborative research on effective countermeasures.

This study provides insights into the prevalence of local TB resistance over the past 8 years. By integrating analyses of phenotypic and molecular resistance mechanisms, regional differences in resistance patterns across various administrative divisions of the city have been examined. It should be noted that, although in-depth sequencing was not performed to verify the specific locations of the drug-resistant sites, and due to certain limitations, we were unable to uncover other drug resistance mechanisms apart from mutations of standard hotspot drug-resistant genes, our findings contribute to the exploration of disparities in medical resource distribution, patient management, and public health awareness across regions, thereby identifying potential factors driving drug resistance variations and informing strategies for improving public health services. The study also enhances the understanding of local DR-TB characteristics, strengthens regional cooperation and exchanges, and promotes technological advancements in TB prevention and control. Additionally, the study supplements the epidemiological data on TB in China and, to some extent, contributes to the global dataset on this disease.

## Conclusion

Regional variations and complex drug resistance patterns continue to pose significant challenges in the fight against TB. The temporal prevalence pattern of local DR-TB shares similarities with findings from domestic and international studies while exhibiting distinct regional characteristics. Resistance can arise through mechanisms beyond common genetic mutations, and MDR/PDR-TB resistance is no longer merely an accumulation of mutations at hotspot loci. This intricate resistance mechanism further complicates local TB control efforts. Continuous updates on the epidemiology of drug-resistant TB are essential to assess the scale of TB transmission in the region accurately and to develop more effective and scientifically informed anti-TB strategies.

## Data Availability

The original contributions presented in the study are included in the article/Supplementary material, further inquiries can be directed to the corresponding authors.
